# Complete Genome Sequence of the Subcluster C1 Mycobacteriophage LordLeafolot

**DOI:** 10.17912/micropub.biology.001992

**Published:** 2026-03-09

**Authors:** Jenna Cagle, MacKenzie Kral, Lauryn Carrington, Rachel Catoni, Olivia Cohn, Heidi Hellenbrand, Jessica Trayhan, Adrian Anderson, Lauren Bailey, Elizabeth Bloomer, Emily Burke, Beatriz Carvalho Mulato, Shiraz Cohen, Noah Daniels, Nicole Dear, Ashley DiBlosi, Caitlin Donello, Emma Dunn, Elizabeth Eisenmesser, Kelly Ellis, Cienna Gewirtzman, Truc Huynh, Henry Keefer, Jonah Litz, Anna Grace Loftis, Ciana Massaria, Kelly Matera, Victoria Matteo, Tanya Montejano, Katherine Morand, Carey Myers, Tracy Nelson, Madelyn Nero, Riya Omar, Erica Owens, Jacqueline Patrick, Claudia Pilgrim, Sarah Porter, Raphaelle Ptasienski, Jordan Roberts, Balor Schnarwyler-Harold, Emma Stacy, Evan Villamor, Jessica McCoy

**Affiliations:** 1 Biology, College of Charleston, Charleston, SC 29412

## Abstract

The complete genome of LordLeafolot, a subcluster C1 virus displaying the myovirus morphology and infecting *Mycobacterium smegmatis* mc²155, is 155,266 bp and encodes 235 predicted proteins, 32 tRNAs, and one tmRNA. The lack of identifiable integrase and repressor genes, along with failed attempts to establish lysogeny, indicates that LordLeafolot has a strictly lytic life cycle.

**Figure 1. Transmission electron micrograph of the C1 mycobacteriophage LordLeafolot:**
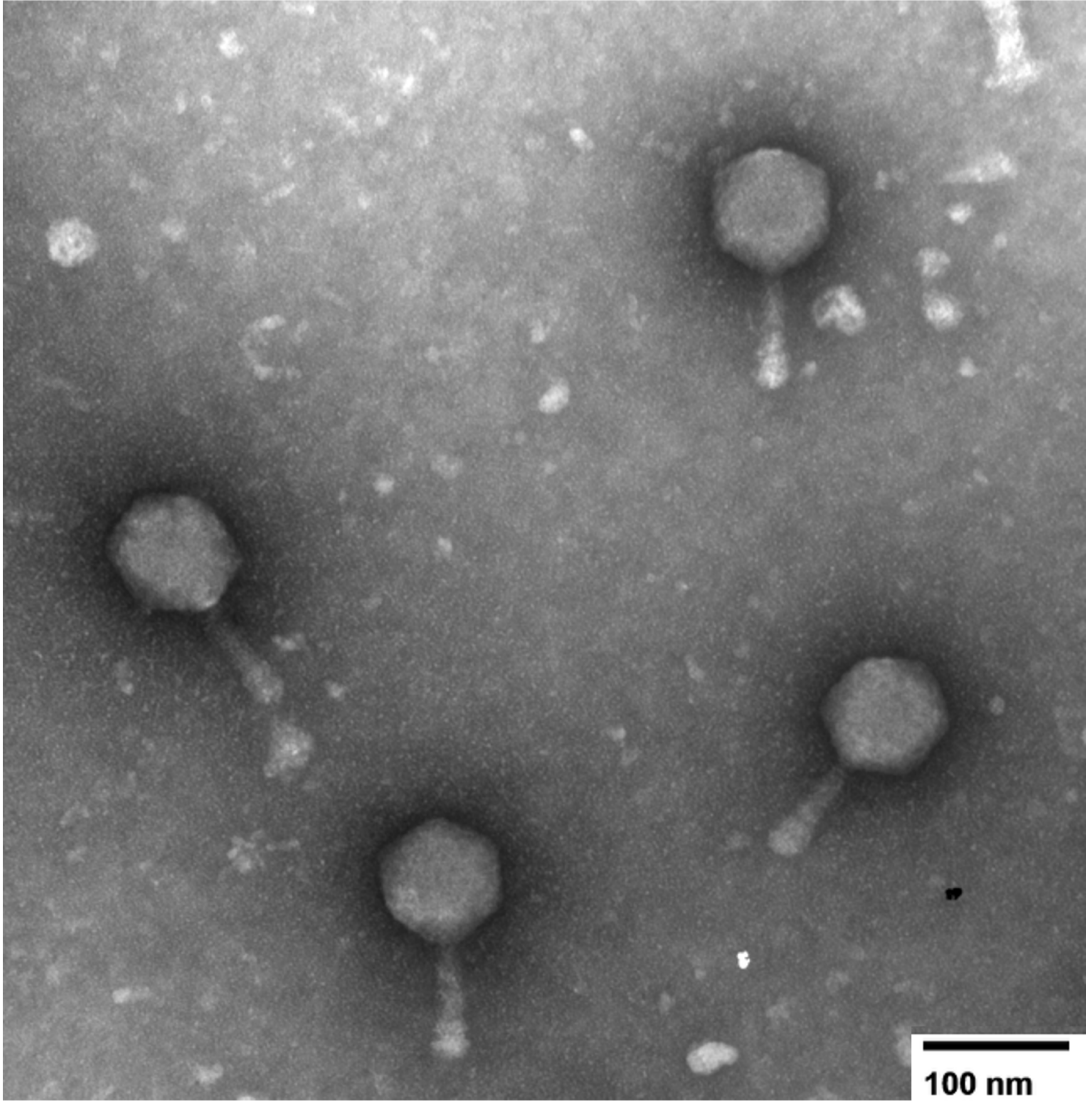
Phage particles from a lysate were negatively stained with 1% uranyl acetate and imaged using a JEOL JEM-1010 transmission electron microscope (TEM). Micrograph was acquired at 150,000× magnification. Scale bar = 100 nm.

## Description

Bacteriophages are emerging as practical alternatives to antibiotics for treating multidrug-resistant bacterial infections. Disseminated *Mycobacterium abscessus* infections have been successfully treated using phages propagated on the nonpathogenic host *Mycobacterium smegmatis* (Dedrick et al., 2023; Hatfull et al., 2022). Here, we present the complete genome sequence and characterization of LordLeafolot, a double-stranded DNA, tailed phage isolated from soil in Charleston, South Carolina, USA, and propagated in *M. smegmatis* . C1 phages are particularly interesting because their large genomes encode extensive sets of tRNAs.

Soil samples were collected from a landscaped campus site (32°47’01.5” N 79°56’19.2” W) and processed using standard methods (Poxleitner et al., 2018). Samples were washed in 7H9 liquid medium with shaking for 3 h at 37°C, the wash passed through a 0.2 μm filter to remove bacteria and debris, and the filtrate inoculated with *M. smegmatis* mc²155, followed by shaking at 250 rpm for 72 h. The resulting culture was refiltered and the filtrate plated in top agar overlay with *M. smegmatis.* A single plaque was picked and subjected to two rounds of plaque purification to ensure a clonal phage population. In top agar overlay assays, purified LordLeafolot produced clear plaques within 24 h. Transmission electron microscopy revealed a myovirus morphology with a contracted tail (108.75 ± 7.39 nm; n = 4) and an isometric capsid (65.00 ± 3.54 nm; n = 4) (Figure 1).

Genomic DNA was extracted from filtered lysate using the Promega Wizard DNA Clean-Up Kit and prepared for sequencing with the NEB Ultra II FS Library Kit. Sequencing on the Illumina NextSeq 1000 platform generated approximately 2.92 million 300-bp paired-end reads. Raw reads were trimmed with cutadapt 4.7 (using the option: –nextseq-trim 30) and filtered with skewer 0.2.2 (using the options: -q 20 -Q 30 -n -l 50) prior to assembly. Genome assembly was performed using Newbler v2.9 (Miller et al. 2010) with default parameters, and completeness was assessed with Consed v29. The complete genome is 155,266 bp circularly permuted, assembled with 2579-fold coverage, and has a G+C content of 64.7%, which is slightly lower than that of the host genome (67.4%) (Mohan et al. 2015). LordLeafolot was assigned to cluster C, subcluster C1, based on gene content similarity of at least 35% to phages in the Actinobacteriophage database, phagesDB ( https://phageDB.org ; Pope et al., 2017; Russell and Hatfull, 2017).

Genome annotation was performed using PECAAN (v20221109; Rinehart et al., 2016), incorporating predictions from Glimmer v3.02 (Delcher et al., 2007), GeneMark v4.28 (Besemer & Borodovsky, 2005), Starterator v558 (http://phages.wustl.edu/starterator/), Phamerator v3 (Cresawn et al., 2011), HHPred v3.1 (Söding et al., 2005), TMHMM v2 (Hallgren et al., 2022), and BLASTp v2.13.0 (Altschul et al., 1990). BLASTp searches were performed against the non-redundant and Actinobacteriophage databases v581. HHPred analyses used PDBmmCIF70, Pfam-A v37, SCOPe v2.08, and NCBI Conserved Domain databases. Transfer RNAs were identified with Aragorn v1.1 in DNA Master (Pope & Jacobs-Sera, 2018), Aragorn v1.2.38 (Laslett & Canback, 2004), and tRNAscan-SE v2.0.6 (Lowe & Eddy, 1997). Putative transmembrane domains were predicted using TMHMM v2 (Hallgren et al., 2022). Analyses were conducted using default parameters.

The completed LordLeafolot genome encodes 268 predicted genes, including 235 protein-coding genes, 32 tRNAs, and one transfer-messenger RNA (tmRNA). Van den Berg et al. (2023) found that C1 phage-encoded tRNAs may help phages evade host defenses by protecting against anticodon-targeting nucleases. Ross and Doore (2025) tested multiple hypotheses for tRNA function in Shigella phage Sf14 and concluded that tRNAs primarily enhance translation of structural genes. Together, these findings underscore the diversity of phage tRNA functions in C1 phage biology, making these phages useful models for phage-host interactions. Functional assignments were made for 21% (52/235) of protein coding genes, including genes in the lysis cassette (lysin A, lysin B) and holin.

Like other C1 phages, LordLeafolot has a circularly permuted, GC-rich genome with modular architecture. Structural and assembly genes are located in the middle portion of the genome, followed by replication and recombination genes, and then lysis genes near the right end. Most genes are transcribed in the forward direction. No integrase or repressor genes were identified, and attempts to raise lysogens produced negative results, as no mesas or stable lysogenic colonies were observed. Collectively, these observations suggest a strictly lytic lifecycle.

**Nucleotide Sequence Accession Numbers**

The genome sequence of LordLeafolot was submitted to GenBank (Accession No. PV876936), and the raw sequencing reads are deposited in the Sequence Read Archive (SRA No. SRX26785851).
